# Acacetin Protects Mice from *Staphylococcus aureus* Bloodstream Infection by Inhibiting the Activity of Sortase A

**DOI:** 10.3390/molecules21101285

**Published:** 2016-09-26

**Authors:** Chongwei Bi, Xiaoyun Dong, Xiaobo Zhong, Hongjun Cai, Dacheng Wang, Lin Wang

**Affiliations:** 1 College of Animal Science, Jilin University, Changchun 130062, China; bicwei@gmail.com (C.B.); 15043029208@163.com (X.Z.); 2 Department of Pharmacology, College of Basic Medical Science, Jilin University, Changchun 130062, China; banyuexiao@126.com; 3 College of Animal Science and Technology, Jilin Agricultural University, Changchun 130062, China; chj6788571@163.com; 4 Key Laboratory of Zoonosis Research, Ministry of Education/Institute of Zoonosis/College of Veterinary Medicine, Jilin University, Changchun 130062, China

**Keywords:** *Staphylococcus aureus*, sortase A, acacetin, inhibitor, renal abscess

## Abstract

*Staphylococcus aureus* (*S. aureus*) is a major cause of infection in hospitals and communities. Widespread dissemination of multi-drug resistant *S. aureus* is a serious threat to the health of humans and animals. An anti-virulence strategy has been widely considered as an alternative therapeutic approach. Inhibitors of virulence factors are able to treat *S. aureus* infections without influencing the growth or viability of bacteria and rarely lead to bacterial resistance. Sortase A (SrtA) is a membrane-associated cysteine transpeptidase that catalyzes up to 25 surface proteins that covalently bind to cell wall peptidoglycans. In *S. aureus*, most of these surface proteins have been identified as important virulence factors that are vital in bacterial pathogenesis. In the present study, we show that acacetin, a natural flavonoid compound, inhibits the activity of SrtA in *S. aureus* (IC_50_ = 36.46 ± 4.69 μg/mL, 128 μM) which affects the assembly of protein A (SpA) to cell walls and reduces the binding of *S. aureus* to fibrinogen (Fg). The mechanism of the interaction between acacetin and SrtA were preliminarily discussed using molecular dynamics simulations. The results suggested that acacetin adopted a compact conformation binding at the pocket of the SrtA via residues Arg-139 and Lys-140. By performing an animal infection model, we demonstrated that acacetin was able to protect mice from renal abscess formation induced by *S. aureus* and significantly increased survival rates. Taken together, these findings suggest that acacetin may be a promising candidate for the development of anti-*S. aureus* drugs.

## 1. Introduction

Anti-virulence strategies have been proposed recently to combat the increasing drug resistance caused by conventional antibiotics combined with a salient lack of research and development of new antibiotics to counteract bacterial pathogens [1]. Many surface proteins of Gram-positive bacteria are thought to be important virulence factors because these surface proteins play key roles in multiple aspects of pathogenesis, such as immune evasion, adhesion and invasion of host cells [2,3,4]. These proteins are covalently linked to the peptidoglycan molecules that compose the cell wall by sortase enzymes [5,6].

SrtA from *S. aureus* (Sa-SrtA) has been extensively studied since its discovery in 1999 [7,8]. Several well-proven virulence factors are wall anchored by Sa-SrtA, including SpA, the fibrinogen-binding clumping factors ClfA and ClfB, and the fibronectin-binding proteins FnbA and FnbB [9,10]. Additionally, SrtA is not essential for the growth and viability of bacteria. Therefore, inhibiting SrtA would be less likely to induce selection pressure, thereby reducing the possibility of resistance developing. In addition, there are no SrtA homologues in eukaryotes with closest function; therefore, inhibiting SrtA is unlikely to result in particularly severe side effects [11,12]. These characteristic features make SrtA a potential target for the treatment of *S. aureus* infection with promising pharmaceutical prospects.

Earlier work identified *srtA* mutants that failed to anchor proteins to the cell wall peptidoglycans [13], displayed great attenuation of virulence and were incapable of leading to sepsis or the formation of abscesses in staphylococcal-infected mice models [9,14,15,16]. Subsequent to the discovery above, the search for and development of SrtA inhibitors has been a hot spot in the research into antimicrobial agents, and several candidates have been investigated [17,18,19]. 

Several strategies have been developed for the identification and characterization of new SrtA inhibitors. The most widely used strategies include screening of natural product or small compound libraries based on enzyme activity, screening based on molecular modelling, pharmacophore hypotheses, 3D-QSAR models and virtual screening, which led to the discovery of natural and chemically synthesized products (reviewed in [17]). In a previous study in our lab, the natural products quercitrin [20] and chlorogenic acid [21] were found to be potent SrtA inhibitors (IC_50_ = 72 μM and 96 μM, respectively). Accordingly, this paper aimed to enlarge the screening range, select a new natural compound with anti-virulence capacity in vivo and in vitro, clarify the mechanisms, and provide a new lead compound or drug candidate for the treatment of *S. aureus* infection.

**Figure 1:**
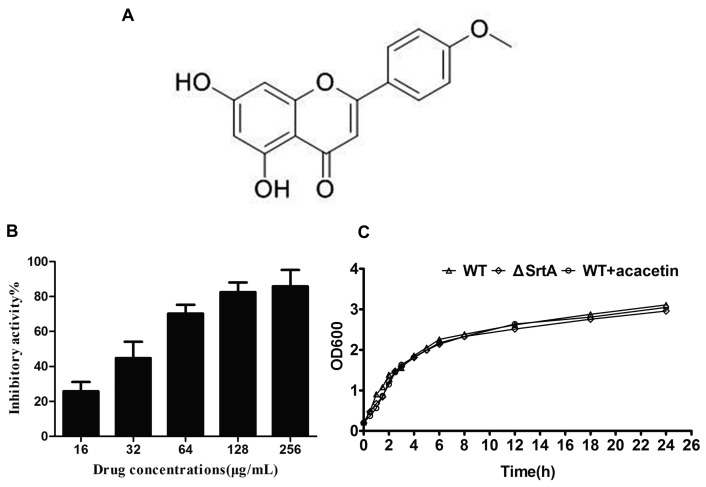
(**A**) Structure of acacetin; (**B**) Inhibitory activity of acacetin (gradient concentrations) against *S. aureus* SrtA in vitro; (**C**) Growth curves of *S. aureus* ATCC25904 (WT) with or without acacetin (256 μg/mL) addition and ΔSrtA.

By performing the FRET assay, acacetin emerged as the best candidate among 50 types of natural compounds with diverse structures that we screened. Acacetin (Figure 1A; IUPAC name: 5,7-dihydroxy-2-(4-methoxyphenyl)chromen-4-one) is an O-methylated flavone which is found in *Robinia pseudoacacia*, *Chrysanthemi indici* Flos, *Tunera diffusa* and *Betula pendula* [22,23].

Acacetin exhibits outstanding anti-peroxidative and anticancer activities and is effective on various cancer cell lines, including lung, leukaemia, prostate, and breast cancer [24,25,26,27]. The anti-arthritic and anti-inflammatory effects of acacetin have also been confirmed in vitro [28]. In particular, a recent research reported that acacetin isolated from the leaves of *Combretum vendae* was active against *S. aureus* [29]. In the present study, we evaluated the anti-*S. aureus* efficacy of acacetin in inhibiting the activity of SrtA by using well-established models in vitro, ex-vivo and in vivo. We further investigated the interaction mechanism of acacetin-SrtA complex using a molecular dynamics simulation study.

## 2. Results

### 2.1. Acacetin Inhibits SrtA-Catalyzed Transpeptidation

The inhibitory activity of acacetin against SrtA was determined using a FRET method [13,30]. A fluorescent peptide substrate (Dabcyl-QALPETGEE-Edans) of SrtA was constructed to monitor the change of the fluorescence during SrtA catalysis. The results were calculated as the inhibition rate, which was expressed as a percentage. Among the tested compounds, we found that acacetin, which is a natural bioflavonoid, had significant catalytic inhibition on SrtA. The detailed results show that the activity of SrtA was blocked by acacetin in a dose-dependent manner (Figure 1B). Based on the inhibition rate of diverse inhibitor concentrations, we calculated an IC_50_ of 36.46 ± 4.69 μg/mL for acacetin in the inhibition of SrtA.

### 2.2. Acacetin Has No Influence on the Growth of S. aureus

Preliminary experiments showed that the MIC values of acacetin on *S. aureus* (ATCC25904 and ΔSrtA strain) were both greater than 1024 μg/mL. Further, a growth curve was studied to reveal whether the growth states varied after the addition of acacetin. According to the results, the growth rate of *S. aureus* WT and ΔSrtA strain was analogous to WT + acacetin (256 μg/mL), even when the concentration was seven times its IC_50_ (Figure 1C). This result indicates that acacetin was able to effectively reduce the activity of SrtA at a dosage that was much lower than its MIC.

### 2.3. Acacetin Influences the Assembly of SpA into the Cell Wall

**Figure 2:**
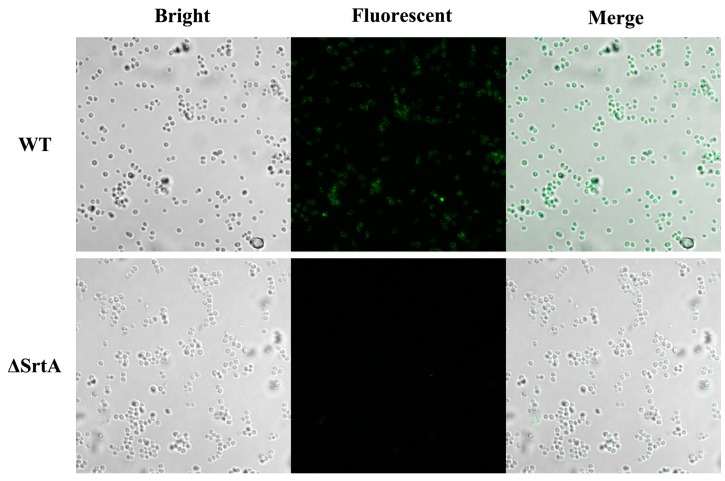

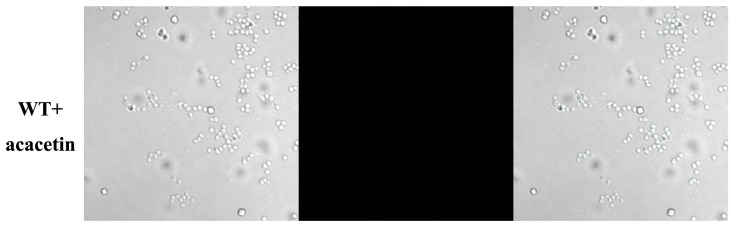
Effects of acacetin on the display of SpA on the surface of *S. aureus* ATCC25904. Binding of FITC-labeled Ig to SpA was viewed via confocal laser-scanning microscope. WT *S. aureus* cells were covered with an obvious green colour. ΔSrtA failed to bind FITC-labeled Ig; the green fluorescence was significantly weaker after acacetin (256 μg/mL) addition.

In *S. aureus*, SrtA anchors more than 20 surface proteins to cell walls, including SpA, which is a multifunctional surface protein that binds to the Fcγ portion of IgG, which in turn is important for immune evasion. Based on its function, we investigated the content variation of SpA in cell wall after the addition of acacetin by staining *Staphylococci* with FITC-labelled goat anti-rabbit IgG. The images obtained from confocal laser-scanning microscope show that *S. aureus* ATCC25904 bound FITC-labelled IgG, which resulted in cells that were covered with an obvious green colour (Figure 2). In contrast, the ΔSrtA strain that lacked SrtA function failed to bind the fluorescent substance. The green fluorescence was significantly weaker when acacetin (256 μg/mL) was added to the broth. These results collectively suggested that acacetin was able to disturb the assembly of SpA into the cell wall by inhibiting SrtA activity.

### 2.4. Acacetin Reduces the Adherence of S. aureus to Fibrinogen

**Figure 3:**
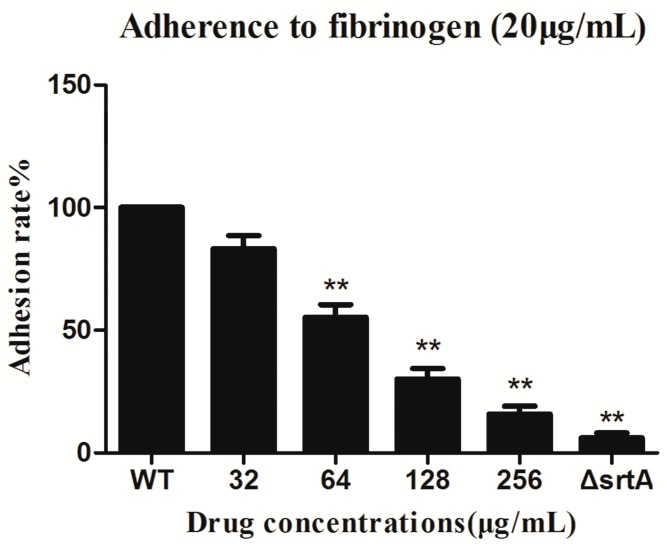
Adhesion rate of *S. aureus* to Fg. Adhesion was significantly inhibited by acacetin in a dose dependent manner. The value of the ΔSrtA was used as a positive control. Three independent experiments were taken to obtain stable result. ∗∗ represents *p* < 0.01 vs. the WT group.

Previous conclusions illustrated that acacetin was able to significantly inhibit the SrtA-catalyzed transpeptidation at sub-MIC concentrations. *S. aureus* expresses up to 25 types of surface proteins that are anchored by SrtA, most of which are essential in the pathogenesis of staphylococcal infection. These surface proteins include clumping factors (ClfA and ClfB), which are vital for the attachment of *Staphylococci* to host Fg, which is the initiation of infection. A virulence reduction of SrtA-deficient *S. aureus* was found in a previous study [9]. One important reason might be that the mutants that lacked SrtA failed to bind Clfs to cell walls, resulting in the loss of Fg-binding function. To test this hypothesis, we performed a Fg-binding assay. The adhesion results showed that the ΔSrtA strain had a minimum binding rate to Fg (6.02 ± 2.09%) (Figure 3), indicating that the Fg-binding function of the ΔSrtA strain was almost completely lost. Treating ATCC25904 (WT) with acacetin significantly reduced the adhesion rates in a concentration-dependent manner compared to no addition (WT). When 32 (112 μM), 64 (224 μM), 128 (448 μM), or 256 (896 μM) μg/mL of acacetin was added, the adhesion rates were 83.35 ± 5.15%, 55.33 ± 5.12%, 30.17 ± 4.7%, and 15.77 ± 3.81%, respectively (Figure 2).

### 2.5. Determination of the SrtA-Acacetin Binding Mechanism

**Figure 4:**
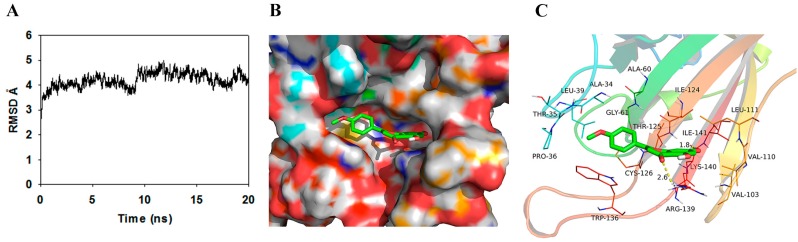
(**A**) Plot of RMSD (in Å) for the SrtA-acacetin complex during 20 ns MD simulation; (**B**) The binding model of SrtA-acacetin complex was shown by PyMoL [http://www.pymol.org/]. The solvent-accessible surface of the protein and the structure of the bound small molecule compound are shown. The surface has been colored to indicate the electrostatic properties from acidic (red) to basic (blue); (**C**) Ribbon diagram of SrtA-acacetin complex was shown by PyMoL.

SrtA-acacetin complex was equilibrated after 20 ns MD simulation, and the plot of root-mean-square deviation (RMSD) (in Ångstroms) of the complex is shown to determine that the system is stabilized during the simulation in Figure 4A. The theoretical binding mode of the acacetin and SrtA is shown in Figure 4B,C. Acacetin adopted a compact conformation binding at the pocket area of SrtA (Figure 4B) and blocked the passway of substrate into the active site. The phenyl group of acacetin was positioned at the hydrophobic pocket, surrounded by the residues Ala-34, Pro-36, Leu-39, Ala-60, Gly-61 and Trp-136 (Figure 4C). Detailed analysis showed that the phenyl group of acacetin formed a CH-π interaction with residue Trp-136. Importantly, the carbonyl “*O*” at the 4-position and the hydroxyl group at the 7-position of acacetin formed two hydrogen bond interactions with the residues Arg-139 (bond length: 2.6 Å) and Lys-140 (bond length: 1.8 Å), respectively, which were the main interactions between acacetin and SrtA. In summary, the above molecular dynamics simulation gave us a rational explanation of the interactions between acacetin and SrtA, which provided valuable information for the further development of SrtA inhibitors.

### 2.6. Acacetin Significantly Increases the Survival Rate of S. aureus-Infected Mice

**Figure 5:**
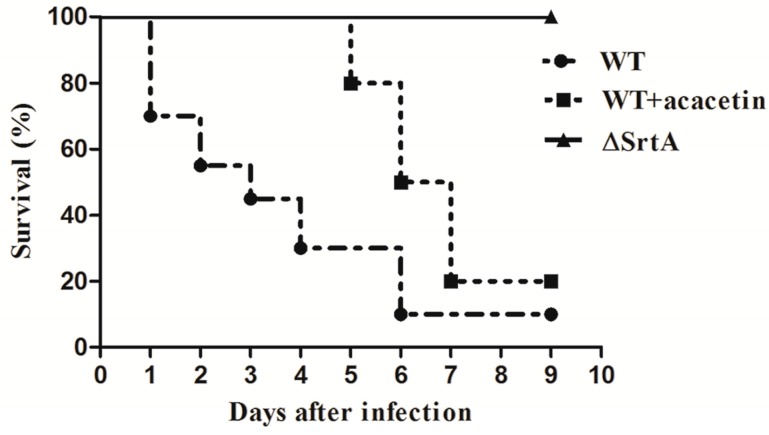
Survival rates of *S. aureus*-infected BALB/c mice. Mice (N = 20) were monitored for 9 days after tail vein injection of 2 × 10^9^ CFU of *S. aureus* (WT) and ΔSrtA. Treatment with acacetin (150 mg/kg/d) was taken after initial infection. Log-rank (Mantel–Cox) test was used for survival statistics. The statistical significance determined as follows: WT vs. WT + acacetin, *p* < 0.05; WT vs. ΔSrtA, *p* < 0.0001.

To investigate the protective effects of acacetin on *S. aureus*-infected mice in vivo, we conducted both fatal and non-fatal infections in the present study. Six days after injection with 2 × 10^9^ CFU of *S. aureus*, which is a lethal concentration for animals, 90% of the mice infected with the WT strain had died. In contrast to the WT strain, the mortality rate of ΔSrtA was still 0% by the observation deadline (Figure 5). The data indicated that SrtA is a considerable virulence factor of *S. aureus*-induced lethal infection. A potential inhibitor of SrtA should have similar performance to become a powerful therapeutic candidate. Drug-treated mice received hypodermic injections of acacetin (150 mg/kg/d) after initial infection and then died of *S. aureus* bacteremia five days after infection. The detailed results suggested that the acacetin-treated mice experienced a significant survival advantage compared to the WT group (Figure 5).

The survival rates were still above 20% on the ninth day after infection. These data indicated that acacetin can significantly prolong the survival of mice with blood-borne infections.

### 2.7. Acacetin Alleviates the Symptoms of S. aureus-Induced Renal Abscess in Mice

**Figure 6:**
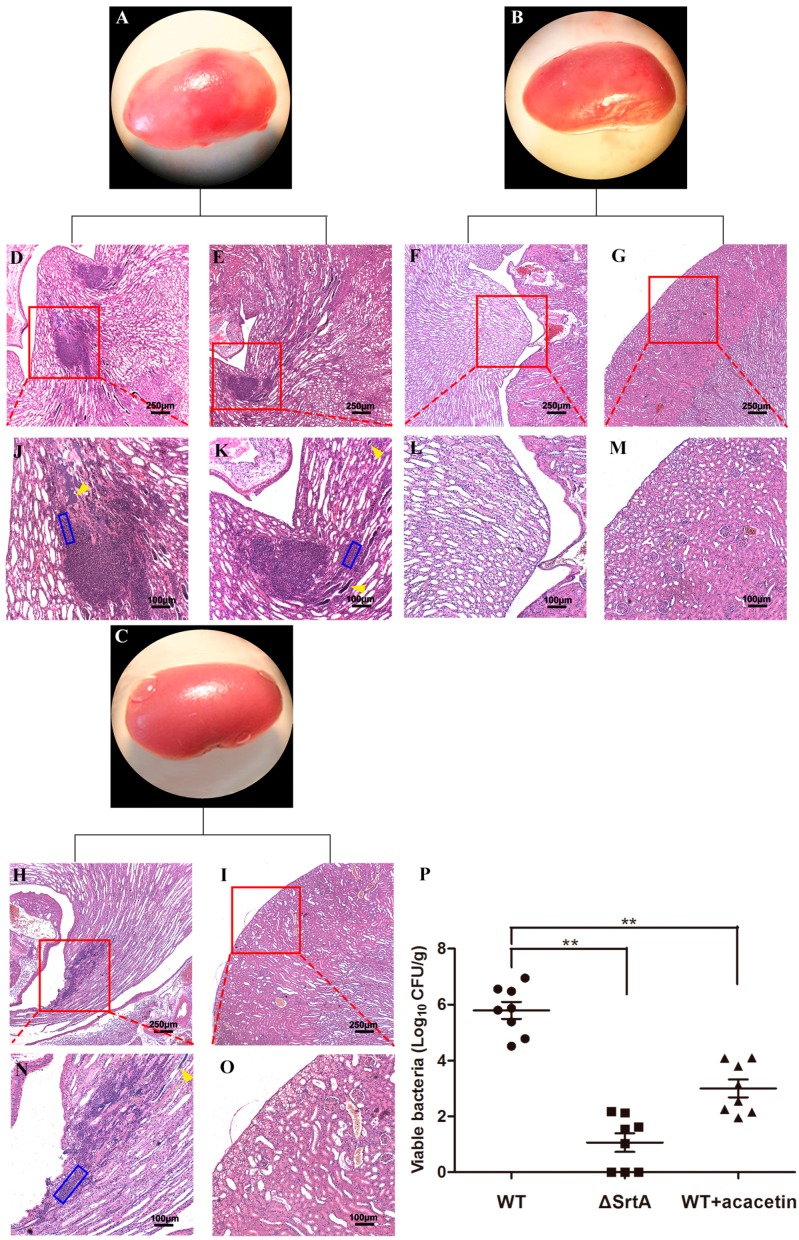
Effects of acacetin on *S. aureus*-induced abscess formation in kidney of BALB/c mice. BALB/c mice (N = 8 per group) was infected with 2 × 10^8^ CFU of *S. aureus* ATCC25904 (WT), ΔSrtA or WT treated with acacetin (150 mg/kg/d). (**A**–**O**) Kidneys were excised for observing surface abscesses (**A**–**C**) or stained with H&E. Histopathology images were acquired with light microscopy at ×100 (**D**–**I**) and ×400 (**J**–**O**). WT infected kidneys exhibited an enclosed *Staphylococci* in the central area (**J**,**K**, yellow arrowheads), surrounded by a large cuff of PMNs (**J**,**K**, blue rectangular box). Acacetin-treated kidneys only had a few *Staphylococci* (**H**, yellow arrowheads) and some slight influxes of PMNs (**H**, blue rectangular box). (**P**) Bacterial burden were obtained from homogenized renal tissues on the 7th day after infection and calculated as log_10_ CFU/g. Student’s *t*-test was performed to determine the statistical significance; ∗∗ represents *p* < 0.01 vs. the WT group.

Bloodstream-infected *S. aureus* typically attaches to specific tissues after evading innate immune defences and then induces the formation of organ abscesses [31]. Previous research suggested that the various surface proteins that are catalyzed by SrtA are vital in *S. aureus* pathogenesis, including SpA, FnbpA and ClfA [14]. By performing survival experiments, we illustrated that acacetin can effectively protect mice from fatal *S. aureus* infection. To study the effect of acacetin on non-fatal *S. aureus*-induced infection, we performed a renal abscess formation experiment by i.v. injecting mice with 2 × 10^9^ CFU of *S. aureus.* In the drug-treated group, acacetin (150 mg/kg/d) was first hypodermically injected 30 min after the initial *S. aureus* infection. Six days after the injection, the mice were euthanized and dissected to excise the kidneys. The left kidneys of the infected mice were homogenized, distributed in PBS, and then plated for the enumeration of CFUs. The CFUs results show that the WT group exhibited a mean of 6.2 × 10^5^ CFU/g in the kidneys, which is remarkably larger than in the ΔSrtA group (11.6 CFU/g) and acacetin-treated group (10^3^ CFU/g) (Figure 6P). The right kidneys were fixed with formalin solution (10%) for histopathology analysis. In macroscopic view, we observed a significant abscess forming on the surface of the WT kidney (Figure 6A), which was larger than that of the acacetin-treated group (Figure 6C). The ΔSrtA kidneys failed to manifest abscess formation and exhibited a normal size and shape (Figure 6B). The results of the pathological section are consistent with the kidney photos. A large area of *S. aureus* is surrounded by polymorphonuclear leukocytes (PMNs), amorphous material and eosinophils in kidneys from WT challenged mice (Figure 6D,E,J,K) [14]. Mice treated with acacetin were prominently alleviated from the symptoms of renal abscess, resulting in a smaller abscess size and fewer PMNs and *Staphylococci* (Figure 6H,I,N,O). These findings suggest that acacetin can protect mice from *S. aureus*-induced infection and reduce the formation of renal abscesses.

## 3. Discussion

*S. aureus* is notorious as a major source of hospital- and community-acquired infections [32]. Bacterial resistance to traditional antibiotics is increasing and becoming more broad-spectrum, leading to the widespread distribution of drug-resistant strains; as a result, the treatment of *S. aureus* infection is becoming increasingly difficult [33]. Anti-virulence strategies have aroused wide interest as alternative approaches for the prevention and treatment of bacterial infections. Because virulence factors are not indispensable for bacterial growth, this strategy might avoid the development of drug resistance.

We have screened potent SrtA inhibitors from various types of natural compounds utilizing the FRET-based cleavage of LPETG as a measure of sortase activity. Acacetin, an *O*-methylated flavone, was able to significantly inhibit SrtA activity both in vitro and ex-vivo without showing a noticeable antimicrobial effect. To our knowledge, only a few articles have reported in vivo therapeutic effects of SrtA inhibitors and validated their inhibitory activity in vitro [34,35]. Although we have reported in detail the activity of chlorogenic acid as an inhibitor of SrtA in vitro and in vivo [21], the flavonoid acacetin, in terms of its structure and mechanisms of interaction with SrtA, is very different. In addition, to date, there are almost no related reports about the antibacterial activity of acacetin, apart from a recent study on acacetin isolated from the leaves of *Combretum vendae* [29]. However, our results indicated that acacetin was able to effectively inhibit the SrtA-catalyzed transpeptidation at sub-MIC concentrations.

We also investigated the protective effects of acacetin on *S. aureus*-infected mice in vivo. The results suggested that acacetin was able to prolong the survival of mice and alleviate the symptoms of renal abscesses (Figure 5 and Figure 6). Molecular docking and molecular simulation were also used in this study to reveal the binding mechanism of acacetin with SrtA. The results indicated that the main interactions between acacetin and SrtA were the carbonyl “*O*” at the 4-position and the hydroxyl group at the 7-position of acacetin, which formed two hydrogen bond interactions with the residues Arg-139 and Lys-140, respectively (Figure 4).

## 4. Materials and Methods

### 4.1. Microbial Strains, Plasmids and Reagents

The reference strains *S. aureus* ATCC25904 (SrtA positive; ClfA positive; coagulase negative; non-haemolytic) was used in this work. The ΔSrtA strain was constructed using ATCC25904 in our previous work [36]. The SrtA expression vector pGSrtA_ΔN59_ was obtained by cloning the SrtA_ΔN59_ sequence into a pGEX-6P-1 vector. *S. aureus* ATCC25904 and ΔSrtA strains were cultured in brain-heart infusion (BHI) solution (Oxoid, Basingstoke, UK) at 37 °C with shaking at 220 rpm. The SrtA_ΔN59_ protein was expressed from pGSrtA_ΔN59_ vector transformed *E. coli* strain BL21 (DE3) and purified by GST affinity chromatography column. Fluorescent-quenched peptide substrate Dabcyl-QALPETGEE-Edans was purchased from Shanghai GL Biochem (Shanghai, China). The acacetin compound was obtained from Chengdu Pufeide Biotech Company (Chengdu, China).

### 4.2. Inhibition of Sortase A Activity

Sortase A activity inhibition assay was performed in 96-well black plates. Fluorescent peptide substrate Dabcyl-QALPETGEE-Edans was used to determine the IC_50_ value. This assay was based on a fluorescence resonance energy transfer (FRET) method that has been described previously [13,30]. Briefly, the reactions contained 10 μM of peptide substrate, 4 μM of SrtA_ΔN59_ protein and the assay buffer (150 mM NaCl, 5 mM CaCl_2_, 0.1% Triton X-100 and 50 mM Tris-HCl, pH 7.5) in a final volume of 300 μL. Increasing concentrations of acacetin and SrtA_ΔN59_ protein were added to the plate and incubated at 37 °C for 1 h; the peptide substrate was then added, and the reaction proceeded at 37 °C for 1 h. The increase in fluorescence intensity was measured using a microplate reader (Infinite^®^ F500, Tecan, Shanghai, China), applying 495 nm as the emission wavelength and 350 nm as the excitation wavelength. Each reaction was repeated three times in this assay to ensure reproducibility.

### 4.3. Minimum Inhibitory Concentrations (MIC) and Growth Curves

The MIC of acacetin against *S. aureus* was determined by broth microdilution on the basis of NCCLS guideline M_31_-A_2_. To plot the growth curves of *S. aureus*, overnight-cultured *S. aureus* was diluted by 1:100 into sterile BHI broth that contained or did not contain appropriate concentrations of acacetin. Absorbance at 600 nm was measured at different intervals of time.

### 4.4. Fibrinogen-Binding Assay

Overnight cultured *S. aureus* ATCC25904 strain was diluted 1:100 into sterile BHI broth and incubated along with variable concentrations of acacetin at 37 °C until *A*_600_ reached 0.5. Positive control was the ΔSrtA strain cultured using the same conditions. Then, all of the cell suspensions were removed, deposited by centrifugation (5000× *g* for 5 min), and suspended in Phosphate buffered saline (PBS) to an *A*_600_ of 1.0. The resuspended cells were added to the wells of a fibrinogen-coated (20 μg/mL bovine fibrinogen, overnight at 4 °C) polystyrene Costar 96-well plate and incubated at 37 °C for 2 h. The liquid was removed. Bacteria were washed with PBS, and 25% (*v*/*v*) formaldehyde was added to fix samples. Adherent bacterial cells were stained with crystal violet solution (12.5 g/L) for 10 min, the wells were washed again with PBS and dried, and the absorbance at 570 nm of disparate samples was subsequently measured. The results were shown using the percentage of the tested group compared to the wild type group.

### 4.5. SpA-Related Fluorescence Analysis

Overnight cultured *S. aureus* was diluted by 1:100 into sterile BHI broth and incubated with or without appropriate concentrations of acacetin at 37 °C until *A*_600_ reached 0.5. The bacteria were washed twice with PBS and then blocked with 2% BSA for 1 h. The bacteria were centrifuged and suspended in PBS containing a 1:100 dilution of FITC-labelled goat anti-rabbit IgG (eBioscience). After 1 h of incubation at room temperature, the *S. aureus* were washed twice with PBS and added to poly-l-lysine-coated glass slides. The fluorescence was observed using a confocal laser-scanning microscope (Olympus, Shanghai, China).

### 4.6. Statistical Analysis

The log-rank test (Mantel-Cox) was used to analyze the survival rate data, and the two-tailed Student’s *t*-test was used to assess the significance of Fg-binding data and bacterial burden data; *p* < 0.05 was considered statistically significant. The molecular dynamics and animal model experimental methods that were used are described in the Supplementary Materials section.

## 5. Conclusions

Overall, our results suggest that acacetin is a potent and promising small molecule anti-SrtA agent which prevents the surface protein Spa from anchoring in the cell wall and prevents the clumping factors (ClfA and ClfB) from playing an adhesion function. The potential therapeutic effect of acacetin on *S. aureus*-induced fatal infection and renal abscesses made acacetin worthy for further development into a novel therapeutic agent. We will improve its physical and chemical properties and biological activities through structural modification and other optimization measures to develop it for use as an alternative or complementary treatment of *S. aureus* infections.

## Supplementary Materials

Supplementary materials can be accessed at: http://www.mdpi.com/1420-3049/21/10/1285/s1.
