# Supplementary material for: Acacetin Protects Mice from Staphylococcus aureus Bloodstream Infection by Inhibiting the Activity of Sortase A
Source: Molecules. 2016 Sep 26;21(10):1285. doi: 10.3390/molecules21101285 (PMC6272931; doi:10.3390/molecules21101285)
Supplement: Supplementary file 1 [file molecules-21-01285-s001.pdf]

Supplementary Materials: Acacetin Protects Mice from *Staphylococcus aureus* Bloodstream Infection by Inhibiting the Activity of Sortase A

Chongwei Bi, Xiaoyun Dong, Xiaobo Zhong, Hongjun Cai, Dacheng Wang and Lin Wang

1. Molecular Dynamics

A molecular docking study was first performed to investigate the binding mode of the acacetin to *Staphylococcus aureus* sortase A using Autodock vina 1.1.2. (The Scripps Research Institute, La Jolla, CA, USA) The three-dimensional (3D) coordinate of the sortase A (PDB ID: 2MLM) was downloaded from Protein Data Bank (http://www.rcsb.org/pdb/home/home.do). The 3D structure of acacetin was drawn by ChemBioDraw Ultra 12.0 and ChemBio3D Ultra 12.0 softwares (PerkinElmer, Waltham, UK). The AutoDockTools 1.5.6 package (http://mgltools.scripps.edu) was employed to generate the docking input files. The search grid of sortase A was identified as center_x: 24.227, center_y: 18.403, and center_z: 11.355 with dimensions size_x: 15, size_y: 15, and size_z: 15. The value of exhaustiveness was set to 20. For Vina docking, the default parameters were used if it was not mentioned. The best-scoring pose as judged by the Vina docking score was chosen and visually analyzed using PyMOL 1.7 software (http://www.pymol.org/).

The Amber 12 [1–3] and AmberTools 13 programs (AMBER Software, San Francisco, CA, USA) were then used for molecular dynamics (MD) simulations of the selected docked pose. CXL was first prepared by ACPYPE [4], a tool based on ANTECHAMBER [5,6] for generating automatic topologies and parameters in different formats for different molecular mechanics programs. Then, the ligand and receptor were prepared by forcefield "leaprc.gaff" and "leaprc.ff12SB", respectively. The system was placed in a rectangular box (with a 10.0 Å boundry) of TIP3P water. The solvated complex was equiled by carrying out a short minimization (500 steps of each steepest descent and conjugate gradient method), 500 ps of heating, and 100 ps of density equilibration with weak restraints using the GPU (NVIDIA® Tesla K20c) accelerated PMEMD (Particle Mesh Ewald Molecular Dynamics) module. At last, 20 ns of MD simulations were carried out.

2. Animal Model of *S. aureus* Infection

Animal experiments were performed using 6- to 8-weeks-old female BALB/c mice following animal care, approved and supervised by the Animal Research Ethics Committee at Jilin University. Overnight cultured *S. aureus* were 1:100 diluted into sterile BHI broth and grown at 37 °C for 3 h. Bacteria were centrifuged, washed twice with PBS, and suspended in BHI broth. *S. aureus* (2×10^9 CFU) were injected into the tail vein of mice to investigate survival rate. For the kidney infection model, animals were challenged by tail intravenous injection of *S. aureus* (2×10^8 CFU). The mouse were euthanized and dissected to excise kidneys on the 7th day after infection. The left kidneys of infected mice were homogenized, distributed in PBS, and then plated for enumeration of CFUs. The right kidneys were fixed with formalin solution (10%) for histopathology analysis. In the treatment group acacetin (150 mg/kg/d) was hypodermically injected at 30 min after injection of *S. aureus*. All animal experiments were performed at least twice to ensure reproducibility.

References

1. Götz, A.W.; Williamson, M.J.; Xu, D.; Poole, D.; le Grand, S.; Walker, R.C. Routine microsecond molecular dynamics simulations with AMBER on GPUs. 1. Generalized Born. *J. Chem. Theory Comput.* **2012**, *8*, 1542–1555.
2. Pierce, L.C.; Salomon-Ferrer, R.; Augusto F de Oliveira, C.; McCammon, J.A.; Walker, R.C. Routine access to millisecond time scale events with accelerated molecular dynamics. *J. Chem. Theory Comput.* **2012**, *8*, 2997–3002.
3. Salomon-Ferrer, R.; Götz, A.W.; Poole, D.; le Grand, S.; Walker, R.C. Routine microsecond molecular dynamics simulations with AMBER on GPUs. 2. Explicit solvent particle mesh ewald. *J. Chem. Theory Comput.* 2013, 9, 3878–3888.

4. Sousa da Silva, A.W.; Vranken, W.F. ACPYPE—AnteChamber PYthon parser interface. *BMC Res. Notes* **2012**, *5*, 367.
5. Wang, J.; Wang, W.; Kollman, P.A.; Case, D.A. Automatic atom type and bond type perception in molecular mechanical calculations. *J. Mol. Graph. Model* **2006**, *25*, 247–260.
6. Wang, J.; Wolf, R.M.; Caldwell, J.W.; Kollman, P.A.; Case, D.A. Development and testing of a general amber force field. *J. Comput. Chem.* **2004**, *2*, 1157–1174.
